# Genetic Mapping and Prediction Analysis of FHB Resistance in a Hard Red Spring Wheat Breeding Population

**DOI:** 10.3389/fpls.2019.01007

**Published:** 2019-08-06

**Authors:** Yuan Liu, Evan Salsman, Jason D. Fiedler, Justin B. Hegstad, Andrew Green, Mohamed Mergoum, Shaobin Zhong, Xuehui Li

**Affiliations:** ^1^Department of Plant Sciences, North Dakota State University, Fargo, ND, United States; ^2^Biosciences Research Laboratory, USDA-ARS Genotyping Laboratory, Fargo, ND, United States; ^3^Department of Crop and Soil Sciences, University of Georgia, Griffin, GA, United States; ^4^Department of Plant Pathology, North Dakota State University, Fargo, ND, United States

**Keywords:** hard red spring wheat, *Fusarium* head blight, genome wide association study, marker-assisted selection, genomic selection

## Abstract

*Fusarium* head blight (FHB) is one of the most destructive diseases in wheat worldwide. Breeding for FHB resistance is hampered by its complex genetic architecture, large genotype by environment interaction, and high cost of phenotype screening. Genomic selection (GS) is a powerful tool to enhance improvement of complex traits such as FHB resistance. The objectives of this study were to (1) investigate the genetic architecture of FHB resistance in a North Dakota State University (NDSU) hard red spring wheat breeding population, (2) test if the major QTL *Fhb1* and *Fhb5* play an important role in this breeding population; and (3) assess the potential of GS to enhance breeding efficiency of FHB resistance. A total of 439 elite spring wheat breeding lines from six breeding cycles were genotyped using genotyping-by-sequencing (GBS) and 102,147 SNP markers were obtained. Evaluation of FHB severity was conducted in 10 unbalanced field trials across multiple years and locations. One QTL for FHB resistance was identified and located on chromosome arm 1AL, explaining 5.3% of total phenotypic variation. The major type II resistance QTL *Fhb1* only explained 3.1% of total phenotypic variation and the QTL *Fhb5* was not significantly associated with FHB resistance in this breeding population. Our results suggest that integration of many genes with medium/minor effects in this breeding population should provide stable FHB resistance. Genomic prediction accuracies of 0.22–0.44 were obtained when predicting over breeding cycles in this study, indicating the potential of GS to enhance the improvement of FHB resistance.

## Introduction

*Fusarium* head blight (FHB) is one of the most destructive diseases of wheat worldwide. The disease is caused by *Fusarium* species and can lead to severe grain yield and quality loss. HRSW is a major class of wheat and is used for the finest baked goods owing to its high protein content and superior gluten quality. In the United States, HRSW is annually planted on over 13 million acres, primarily in the Northern Great Plains. FHB epidemics in HRSW producing areas of the US in the early 1990s resulted in devastating economic losses (Wilcoxson et al., 1992; McMullen et al., 1997) and the disease still frequently threatens wheat production in this region. Controlling FHB through agronomic practices such as crop rotation is challenging due to the wide host range of the *Fusarium* species. Fungicidal control is effective only in a narrow operative window. Therefore, developing FHB resistant cultivars is crucial to prevent the destruction caused by this disease.

*Fusarium* head blight resistance exhibits quantitative variation, suggesting complex genetic architecture. Most public wheat breeding programs in the US rely on phenotypic selection for FHB resistance using field nurseries to evaluate breeding materials. However, disease infection and development is highly dependent on environmental conditions at flowering and early kernel development stages, making effective phenotypic evaluation and selection difficult. In addition, phenotypic evaluation of FHB resistance is time-consuming and demands extensive labor and resources in multiple environment trials, limiting the number of breeding lines that can be evaluated in each breeding cycle. MAS of major QTL could enhance selection efficiency for FHB resistance (Anderson, 2007; Buerstmayr et al., 2009). From previous bi-parental QTL mapping studies, hundreds of QTL for FHB resistance have been identified on all 21 wheat chromosomes (Buerstmayr et al., 2009; Löffler et al., 2009). In previous studies, few QTL were identified for type I resistance (against initial infection of the pathogen) as most previous studies focused on type II resistance (against spread of the pathogen) (Buerstmayr et al., 2009). Many QTL were commonly identified from multiple populations (Liu et al., 2009; Löffler et al., 2009), of which major QTL like *Fhb1* (Bai et al., 1999; Waldron et al., 1999; Anderson et al., 2001; Liu et al., 2006; Rawat et al., 2016; Su et al., 2018), *Fhb2* (Cuthbert et al., 2007; Jia et al., 2018), *Fhb4* (Xue et al., 2010; Jia et al., 2018), and *Fhb5* (Xue et al., 2011; Jia et al., 2018) were finely mapped and/or characterized. The most stable type II resistance QTL *Fhb1* can reduce disease severity by 20–25% on average, depending on the genetic background (Pumphrey et al., 2007), and has been widely integrated into wheat breeding populations through MAS (Anderson, 2007; Buerstmayr et al., 2009; Steiner et al., 2017). However, utilization of other QTL for MAS in wheat breeding has been scarce, due to lack of diagnostic markers or pending validation.

Genome wide association studies (GWAS) enable genetic mapping to be performed in a breeding population, and the identified markers can be used for MAS directly (Pozniak et al., 2012; Wang et al., 2012). Advances in genome sequencing and high-throughput genotyping have facilitated GWAS in wheat, a polyploid species of large genome size (Poland et al., 2012; Wang et al., 2014; Allen et al., 2017; International Wheat Genome Sequencing Consortium [IWGSC], 2018). Numerous GWAS have been conducted for FHB resistance in wheat and confirm its complex genetic architecture (Mirdita et al., 2015b; Arruda et al., 2016a; Wang et al., 2017). Arruda et al. (2016a) performed GWAS for FHB resistance using 273 winter wheat breeding lines from the Midwest and Eastern US, and found that *Fhb1* explained only 8% of phenotypic variation. GWAS using a large panel of elite inbred lines from central European winter wheat found no QTL with large effect, despite broad genetic variation (Mirdita et al., 2015b). Taken together, these observations confirm that many genes with medium or small effects contribute to FHB resistance along with the few well-characterized major QTL.

A simulation study suggested that GS was more efficient for the improvement of complex traits compared to MAS (Meuwissen et al., 2001). In GS, genome wide markers are used to predict breeding values, which are consequently used for selection. Empirical experiments found that prediction ability of FHB resistance using genome wide markers was better than using statistically significant markers only (Jiang et al., 2015; Mirdita et al., 2015a; Arruda et al., 2016b). Several GS studies suggested that GS is promising to enhance FHB resistance improvement in wheat (Rutkoski et al., 2012; Mirdita et al., 2015a; Jiang et al., 2017; Herter et al., 2019a,b).

Diverse resistance donors like Sumai 3, Frontana, etc. have been used for the improvement of FHB resistance in wheat breeding programs including the NDSU HRSW breeding program. Well-characterized major QTL like *Fhb1* and *Fhb5* have been integrated into the breeding pools. The objectives of this study are to (1) investigate genetic architecture of FHB resistance in this breeding population; (2) test if the major QTL *Fhb1* and *Fhb5* play an important role in this population; and (3) assess potential of GS for FHB resistance enhancement in the NDSU HRSW breeding program.

## Materials and Methods

### Plant Materials and Evaluation of FHB Severity

In total, 427 F_9_ breeding lines from 2011-16 advanced yield trials (AYTs) of the NDSU HRSW breeding program, representing six breeding cycles, and 15 check cultivars were evaluated for FHB resistance from 2011 to 2016 (Supplementary Table S1). Each year, a subset of breeding lines from one breeding cycle was evaluated at two locations, Langdon and Prosper, ND. However, the trials from 2013 Prosper and 2016 Langdon were not used because of poor quality of the phenotypic data. Therefore, the 427 breeding lines were actually tested in an unbalanced experiment including 10 trials across 6 years (Supplementary Table S1). The 15 check cultivars were ND2398, ND2710, Barlow, Bolles, Elgin-ND, Faller, Glenn, Howard, ND817, Prosper, RB07, Steele-ND, SY Soren, and Velva. The checks ND2398, ND2710, Barlow, Faller, Glenn, and Prosper were evaluated at all 10 trials at the two locations from 2011 to 2016. In each trial, the experimental design was a randomized complete block design with two replicates. In the field experiment, each line was planted in a hill plot with 15 seeds. Spawn corn kernels were prepared as inoculum. To prepare inoculum, autoclaved corn kernels were infected with a mixture of spores produced separately from 20 *F. graminearum* strains, including ten 3ADON (3-acetyl deoxynivalenol) producers and ten 15ADON (15-acetyl deoxynivalenol) producers, collected from fields in North Dakota (Puri and Zhong, 2010), according to the procedure described by Zhang et al. (2008). The spawn corn kernels were applied to the FHB nurseries at a rate of approximately 0.20 kg/m^2^ starting at the boot stage, and repeated every 2 weeks until all wheat accessions completed anthesis. The nurseries were overhead misted for 5 min in 15-min intervals for 12 h daily (4:00 p.m. to 4:00 a.m.), until 14 days after anthesis of the latest lines. Eight to twenty spikes from each hill plot were visually scored at 21 days post anthesis using a visual scale (0, 7, 14, 21, 33, 50, 66, 75, 90, and 100, all in percentage) (Stack and McMullen, 1995). Disease severity was calculated as the average percentage of infected spikelets.

### Molecular Marker Genotyping

The 427 elite breeding lines and 12 of the 15 check cultivars were genotyped using GBS. DNA was isolated with the Wizard Genomic DNA Purification Kit (A1125; Promega) per the manufacturer’s instructions and quantified with a Quant-iT PicoGreen dsDNA assay kit (P7589; Thermo Fisher Scientific). GBS libraries were constructed based on the protocol of Poland et al. (2012) with minor modifications. Briefly, 200 ng of DNA for each line was digested with *Pst*I and *Mse*I, and then ligated to a common adapter and a barcoded adapter unique to each sample. Equal volumes of the ligated products were pooled and purified with the QIAquick PCR purification kit (28104; QIAGEN) for PCR amplification. For the PCR amplification, 50 ng of template DNA was mixed with NEB 2X Taq Master Mix and two primers (5 nmol each) in 200 μL total reaction volume and PCR amplified for 18 cycles with 10 s of denaturation at 98°C, followed by 30 s of annealing at 65°C, and finally 30 s extension at 72°C. The PCR product was cleaned using a QIAquick PCR purification kit. The library was sequenced on an Illumina HiSeq 2500 to generate single-end, 100-bp reads at the Genomic Sequencing and Analysis Facility at the University of Texas Southwestern Medical Center at Dallas, Texas. All sequences were submitted to the National Center for Biotechnology Information Short Read Archive (experiment #SRP144046). SNP discovery and genotype calling was performed using the TASSEL-GBS pipeline (Glaubitz et al., 2014) with the *Triticum aestivum* IWGSC RefSeq v1.0 as the reference genome (International Wheat Genome Sequencing Consortium [IWGSC], 2018). SNP markers were filtered with an individual read depth greater than 1, MAF greater than 0.05, and missing data less than 50%. Filtering SNPs yielded 102,147 SNP markers (Supplementary Table S1). Missing values were imputed with LD-KNNi method (Money et al., 2015) implemented in TASSEL v.5 (Bradbury et al., 2007).

Additionally, one SNP marker (*IFA-FM227*, Schweiger et al., 2016) linked to the major QTL *Fhb1* and one SNP marker (barc186-80018, unpublished) linked to the major QTL *Fhb5* were genotyped for the spring wheat breeding lines and check cultivars. Primer sequences for IFA-FM227 were as follows: FAM: GGCGTCGGCGATCCTGCTTA; HEX: GGCGTCGGCGATCCTGCTTAT; Common: CGTCGT CGGCCGCGGGTT. Primer sequences for barc186-80018 were as follows: FAM: GTAGTGATCCAAAGAAATAAAGGAGAT; HEX: GTAGTGATCCAAAGAAATAAAGGAGAG; Common: GTGACAAGTTATAGGTAAGGTCTCCAT. Each SNP was assayed using the KBiosciences Competitive Allele-Specific PCR genotyping system (KASP). Briefly, genotyping reactions were performed in 384-well plates on a Procycle thermocycler (Life) in 4 μL reactions containing two allele-specific primers, one common reverse primer, 1 x KASPaR v4.0 SNP Mastermix (LGC Genomics), and 36 ng of gDNA. After an initial denaturation step of 15 min at 94°C, PCR amplification reactions consisted of 10 cycles of 20 s denaturation at 94°C, followed by 60 s at a touchdown annealing/elongation at 65-57°C (decreasing 0.8°C each cycle), then 29 cycles at 20 s denaturation at 94°C, followed by annealing/elongation 60 s at 57°C. Fluorescence of PCR products was measured on a Roche Light Cycler^®^ 480 and accompanying software (v 1.51) was used to distinguish clusters and call genotypes.

### Phenotypic Data Analysis

The number of breeding lines per trial ranged from 31 to 112 (Supplementary Table S1). Two-stage analysis of phenotypic data was performed. In the first stage, best linear unbiased estimators (BLUEs) were estimated for all breeding lines within each individual trial using R package lme4 (Bates et al., 2014; R Development Core Team, 2018). The model was

[1] $$y=\mu +g_{i}+r_{j}+\varepsilon_{i\,j}\,$$

where y is the vector of unadjusted phenotypes, μ is the overall mean, *g*_*i*_ is the fixed effect of the *i*_*th*_ genotype and *r*_*j*_ is the random effect of the *j*_*th*_ block. To identify outlier trial, we estimated broad-sense heritability (*H*^2^) for each individual trial. All factors were considered as random. The variance components of error (σ^2^) and genotype (σ^2^_*g*_) were estimated. The broad-sense heritability was estimated as σ^2^/(σ^2^ + σ^2^_*g*_) and was used to eliminate trials with a *H*^2^ score < 0.1.

In the second analysis stage, we estimated BLUEs of breeding lines across all 10 trials using the R package lme4 (Bates et al., 2014). The model was

[2] y=*μ+gi+tj+εi⁢j

where y^*^ represents the estimated BLUEs of breeding lines within each individual trial calculated in the first stage, μ is the overall mean, *g*_*i*_ is the random effect of the *i*_*th*_ genotype and *t*_*j*_ is the random effect of the *j*_*th*_ trial. The estimated BLUEs were further used in GWAS to identify major QTL and in genomic prediction analysis.

A relationship matrix was calculated using the 102,147 SNP markers with the R package rrBLUP (Endelman, 2011). Then, additive variance components (*Va*) and error variance component (*Ve*) were calculated with the mixed.solve function in the package rrBLUP (Endelman, 2011). Genomic heritability (*h*^2^) was calculated as *Va*/(*Va* + *Ve*).

### Marker-Trait Association Analysis

Principal component analysis (PCA) was conducted with the 102,147 SNPs to assess population structure using TASSEL v.5 (Bradbury et al., 2007). SNP-based GWAS was performed using TASSEL v.5 (Bradbury et al., 2007). Based on the Scree plot (Supplementary Figure S1), the first three PCs were chosen as covariates to capture population structure in association analysis. A centered kinship (K) matrix was calculated based on the 102,147 SNPs using TASSEL. A linear mixed model including population structure and kinship matrix was used to test marker-trait association. False discovery rate (FDR) was calculated from p-values using the R function p.adjust (method = fdr) (Benjamini and Hochberg, 1995). Significance of marker-trait association is defined by FDR as a *q* value smaller than 0.1.

### Development and Validation of Genomic Prediction Models

Genomic prediction was evaluated with the statistical model rrBLUP. The rrBLUP model was constructed using R package rrBLUP (Endelman, 2011). Prediction accuracies were first validated using five-fold cross-validation, in which 80% of individuals were randomly selected as the training population and the remaining 20% of individuals were used to validate the genomic prediction accuracy. Genomic prediction accuracy was estimated as the Pearson correlation (*r*) between genomic estimated breeding values (GEBVs) and BLUPs of phenotypic values. Random sampling of training and validation sets was repeated 100 times and the mean of *r* divided by the square root of the estimated heritability was defined as the genomic prediction accuracy.

To test prediction accuracy across breeding cycles, we developed prediction models using a training population that includes five breeding cycles to predict breeding lines from the remaining breeding cycles, e.g., to predict 2012 breeding lines, the five breeding cycles tested in the other 5 years (2011, 2013–2016) were used as training population.

Because genetic relationship between training population and validation population can affect prediction accuracy, we calculated genetic relationships between training population and validation population based on identity-by-state similarity (proportion of shared alleles) between all individuals using R package “snpRelate” (Zheng et al., 2012).

To evaluate the effect of marker number on prediction accuracy, we randomly sampled 100, 500, 1000, 1500, 2000, 2500, 3000, 3500, 4000, 4500, and 5000 markers to develop prediction models and validate their prediction accuracies. Random sampling for each subset was repeated 500 times and the mean of the Pearson correlations between GEBVs and BLUPs was defined as the genomic prediction accuracy.

## Results

### Genetic Diversity of the Elite Spring Wheat Lines and Their Reaction to FHB

Broad-sense heritability was estimated for each individual trial and ranged from 0.21 to 0.67. No outlier trials were identified at threshold of *H*^2^ < 0.1. BLUEs of FHB severity were estimated for the 427 elite spring wheat lines and 15 check cultivars using the unbalanced phenotypic data collected from all 10 field trials at two locations across 6 years. Among the 15 checks, the resistant check variety ND2710 showed the lowest FHB severity (15.9%) while the susceptible check variety ND2398 had the highest disease severity (75.8%). Among the 427 breeding lines, FHB severity ranged from 6.0% to 83.1% and broad variation was found in all six breeding cycles (Table 1). In total, 102,147 markers were obtained with missing values less than 50%. MAF of the 102,147 markers averaged 0.19, ranging from 0.05 to 0.5 (Supplementary Figure S2). PCA was performed and the PC1 and PC2 explained 6.9 and 6.0% of total variation, respectively. Scatter plots of PC1 against PC2 for the 427 lines indicated there was no clear population structure and breeding lines from different breeding cycles were intermixed (Supplementary Figure S3).

**TABLE 1:** Means and ranges of FHB severity for the breeding lines from six breeding cycles and 15 checks.

		**FHB severity (%)**	
**Breeding cycle**	**Number of lines**	**Mean**	**Range**
2011	112	36.3	21.1~83.1
2012	72	37.0	17.8~55.0
2013	31	32.6	17.7~47.9
2014	66	36.7	20.1~52.2
2015	65	33.5	10.3~56.5
2016	81	29.4	6.0~54.1
Check	15	39.5	15.9~75.8

### Marker-Trait Association

**FIGURE 1:**
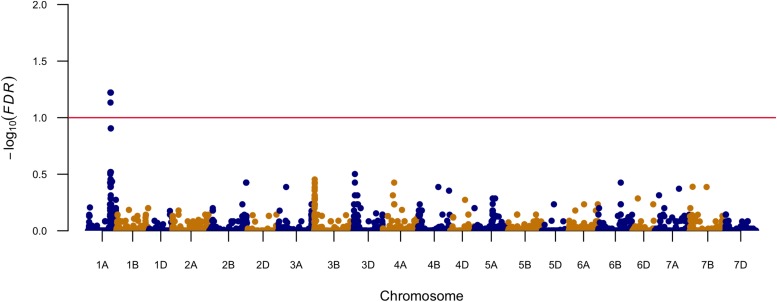
Manhattan plot that displays genomic regions associated with FHB severity.

Marker-trait association was conducted with a linear mixed model including population structure and kinship matrix. Significant marker-trait association was determined by FDR as a *q* value smaller than 0.1. The Manhattan plot is shown in Figure 1. In total, six SNPs significantly associated with FHB resistance were identified on chromosome arms 1AL (Figure 1 and Table 2). The most significant markers (S1A_477852878 and S1A_477852881) explained 5.3% of the total phenotypic variation (Table 2). Over 81% of the 439 tested lines had the favorable allele for the two most significant markers at this locus (Table 2). The favorable allele alone could reduce 7.5% of FHB severity (Table 2).

**TABLE 2:** Information of the SNP markers significantly associated with FHB severity and the SNP markers linked to the major QTL *Fhb1* and *Fhb5*.

**Marker**	**Chr^a^**	**Position^b^**	***p*-value**	**FDR**	***R*^2^ (%)^c^**	**Allele**	**Effect (%)^d^**	**Obs^e^**
S1A_471476097	1A	471476097	2.47E-06	0.06	5.3	A/g	−7.49	348/83
S1A_471476711	1A	471476711	2.52E-06	0.06	5.3	G/t	−7.48	349/83
S1A_471700659	1A	471700659	4.32E-06	0.07	5.0	C/t	−7.36	350/81
S1A_471719877	1A	471719877	2.94E-06	0.06	5.2	G/a	−7.45	348/82
S1A_477852878	1A	477852878	2.63E-06	0.06	5.3	C/t	−7.52	353/78
S1A_477852881	1A	477852881	2.63E-06	0.06	5.3	A/g	−7.52	353/78
IFA-FM227	3B	8848534	0.0003	0.49	3.1	A/g	−5.25	140/289
barc186-80018	5A	350267183	0.1463	0.99	0.5	A/g	−2.27	111/324

*^*a*^Chromosome. ^*b*^Physical position of SNP marker on wheat reference genome. ^*c*^The percentage of total variation explained by the marker. ^*d*^The estimated additive effect of the favorable allele. ^*e*^Number of breeding lines observed for each marker allele.*

### Frequencies and Effects of the Molecular Markers Linked to *Fhb1* and *Fhb5*

We genotyped one SNP marker linked to the major QTL *Fhb1* and one SNP marker linked to the major QTL *Fhb5*. The marker linked to *Fhb1*, IFA-FM227, was significantly associated with FHB severity at *p*-value less than 0.001 and explained 3.1% of total phenotypic variation (Table 2). In total, 140 (33%) of the 429 lines contained the favorable allele of the major QTL *Fhb1*. The marker linked to *Fhb5*, barc186-80018, was segregated in this population with the favorable allele frequency at 26% (Table 2). However, this marker was not significantly associated with FHB severity at *p* value smaller than 0.05 (Table 2).

### Genomic Prediction

Based on the marker and phenotypic data, genomic heritability (*h*^2^) for the FHB severity was estimated to be 0.43 in this HRSW breeding population. We evaluated genomic prediction for FHB severity with statistical model rrBLUP. Prediction accuracy using fivefold cross validation was 0.35.

**FIGURE 2:**
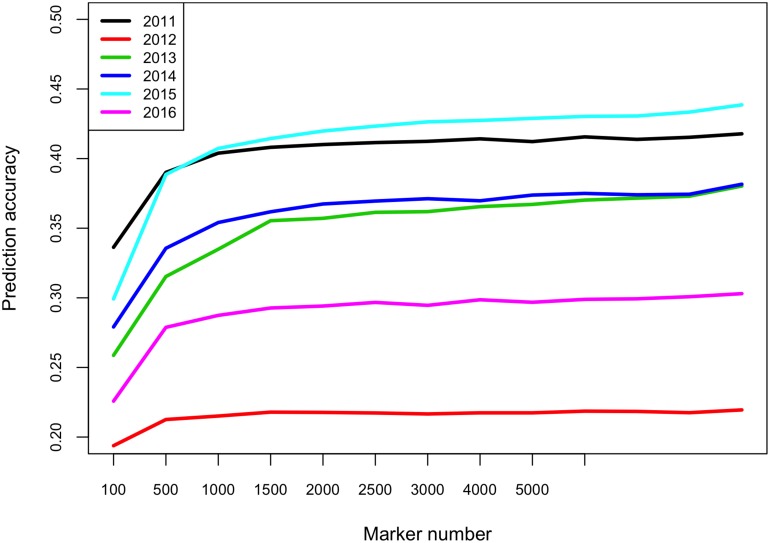
Prediction accuracies for FHB severity across different breeding cycles using varied number of markers. E.g., to predict 2012 breeding lines, the breeding lines evaluated in the other five breeding cycles (2011, 2013–2016) were used as training population. The *x*-axis shows the number of markers in each subset (100, 500, 1000, 1500, 2000, 2500, 3000, 4000, 5000, and 102147 markers).

One potential application of GS is to predict breeding lines from a new breeding cycle and perform pre-selection. To test such prediction ability, we used breeding lines from five of the six breeding cycles as training population to predict breeding lines from the remaining breeding cycle. The prediction accuracies ranged from 0.22 when predicting 2012 breeding lines to 0.44 for predicting 2015 breeding lines (Figure 2). The identity-by-state similarity between all pairs of breeding lines were calculated using the 102,147 markers. The genetic relationships between one breeding cycle and other breeding cycles were calculated based on the identity-by-state similarities between pairs of breeding lines. We found that the genetic relationships between the breeding cycles were similar to the average genetic relationship of all pairs of breeding lines (Supplementary Figure S4) and pairs of breeding lines within a breeding cycle (data not shown). Therefore, genetic relationships between training and validation population did not impact the prediction accuracies, which varied between different breeding cycles. We also evaluated effect of marker number on prediction accuracy. The prediction accuracies increased from 100 markers to 1000 markers but attained a plateau after 1000 markers (Figure 2).

## Discussion

### Genetic Architecture of FHB Resistance in the NDSU HRSW Population

The major QTL *Fhb1* is the most stable type II resistant QTL identified, with a large genetic effect consistently presented in previous bi-parental mapping studies (Anderson et al., 2001; Pumphrey et al., 2007; Buerstmayr et al., 2009; Zhao et al., 2018b). In this study, we evaluated FHB reactions of 427 breeding lines from the NDSU HRSW breeding program, but the marker linked to *Fhb1* showed a relatively small effect, explaining approximately 3.1% of the total phenotypic variation. The varied effects of *Fhb1* have to do with differences in genetic background, tested environments, and/or resistance types evaluated. For example, Bokore et al. (2017) evaluated Sumai 3-derived North American spring wheat breeding lines at two field nurseries in Canada and found that *Fhb1* was more effective at one location than the other and that *Fhb1* was more effective in some families than others. In this study, FHB resistance – measured as severity – was conducted in field nurseries, where FHB resistance assessment is confounded by natural infection in addition to manual inoculation. Different from single-spikelet inoculation, high levels of natural *Fusarium* inoculum can increase infection incidences, mitigating the effectiveness of *Fhb1* and the type II resistance it confers. Zhao et al. (2018b) studied type II FHB resistance using a population derived from a cross between ND2710 and Bobwhite with single-spikelet inoculation, and detected *Fhb1* as major QTL in both greenhouse and field evaluations in North Dakota, however, with *Fhb1* explaining much less phenotypic variation under field conditions. Failing to detect the QTL *Fhb1* for FHB severity under field inoculation was also reported in winter wheat (Herter et al., 2019b). Some studies found that the QTL *Fhb5*, providing type I resistance, was of higher effect than *Fhb1* under field inoculation (Von der Ohe et al., 2010; Miedaner et al., 2019). However, the QTL *Fhb5* was not significantly associated with FHB severity in this study. The discrepancy between this study and the other two studies (Von der Ohe et al., 2010; Miedaner et al., 2019) remains unclear.

We identified one QTL on chromosome arm 1AL in this breeding population through association mapping. A few previous mapping studies also detected FHB resistance QTL on chromosome arms 1AL (Lin et al., 2004; Petersen et al., 2017; Yi et al., 2018). Petersen et al. (2017) identified a QTL related to Type I resistance between 90K-SNP markers IWB29758 and IWB73950 on chromosome arm 1AL using a double haploid population derived from two US soft red winter wheat cultivars. The physical locations of the two markers were 539895992–541895688 bp, determined by BLASTn searches of the primer sequences against the *Triticum aestivum* IWGSC RefSeq v1.0 (International Wheat Genome Sequencing Consortium [IWGSC], 2018). Yi et al. (2018) found a QTL related to FHB severity on chromosome arm 1AL, where the closest 90K-SNP markers were RAC875_c6338_1887 under single-spikelet inoculation and wsnp_CAP12_c2438_1180601 under spay inoculation, respectively. The two markers were located at 350007881 and 39649561 bp on chromosome arm 1AL. The QTL found from our study are distant from those identified from previous studies, suggesting it is a possible novel locus for FHB resistance.

Glenn is a HRSW cultivar with good grain yield and high protein content (Mergoum et al., 2006). Glenn showed high resistance to FHB despite of lacking the favorable allele of *Fhb1* (Mergoum et al., 2006; Bokore et al., 2017); our results confirmed this observation (data not shown). The cultivar Glenn and its derived lines have been widely used as parents in the NDSU HRSW breeding program. A Chinese landrace Haiyanzhong has shown high level of resistance to FHB, and a mapping study using a population derived from Haiyanzhong found that it carried *Fhb2*, *Fhb4*, *Fhb5*, and four other minor QTL (Cai et al., 2016). The accumulation of QTL with medium/minor effects conferring high FHB resistance have been identified in other wheat lines such as PI277012 (Chu et al., 2011; Zhao et al., 2018a), Wanshuibai (Lin et al., 2006), and Frontana (Steiner et al., 2004). Therefore, the high level of FHB resistance observed in Glenn and other breeding lines in this HRSW breeding population is likely a result of numerous genes. The identified QTL explained 5.3% of total phenotypic variation and the genomic heritability estimated with genome wide markers was 0.43, suggesting that there are unidentified genes with small additive effects that also contribute to the FHB resistance. Integrating QTL with major, medium, and minor effects for both type I and type II resistance seems crucial to developing stable FHB resistance under field conditions.

### GS for FHB Resistance Improvement

MAS of major QTL like *Fhb1* has contributed to the development of FHB resistant cultivars in several wheat breeding programs (Anderson, 2007; Steiner et al., 2017). However, phenotypic selection is still routinely utilized in wheat breeding programs due to the complex genetic architecture of FHB resistance. Phenotypic selection of FHB resistance is generally conducted in later generations with replicates because of strong genotype by environment interactions (Anderson, 2007). The 427 breeding lines used in this study were F_9_ breeding lines from 2011 to 2016 AYTs, which is a 3-year experiment. Most of the breeding lines had been evaluated for FHB resistance in at least two prior years using F_7_ or F_8_ generations. Some breeding lines with good grain yield and/or quality traits could have been selected into AYTs even if possessing only moderate FHB resistance. Additionally, it is likely that many F_5_ or F_6_ breeding lines with good FHB resistance and grain yield potential were missed during generational selection due to limited resources for FHB and grain yield evaluation.

In GS, selection is based on the predicted breeding values from genome wide markers. One potential application of GS is to select promising lines from a large number of newly developed early generation breeding lines (i.e., F_5_ or F_6_) into expensive yield trials and therefore improve selection efficiency. For example, GS pre-selection of end-use quality traits in wheat has successfully reduced the number of poor performing lines advanced to expensive yield trials (Guzman et al., 2016). In this study, we found medium prediction accuracy of 0.35 using cross-validation within population. Higher prediction accuracies were observed for FHB resistance from some previous studies (Mirdita et al., 2015a; Arruda et al., 2016b; Galiano-Carneiro et al., 2019). In most of the previous studies, balanced phenotypic data was used where all lines were phenotyped at same set of environments. In this study, phenotypic data for the 439 breeding lines collected from different years and locations were unbalanced, and the estimated BLUEs were adjusted by common checks. Because genotype by environment interaction could not be integrated in the statistics models, bias of the estimated BLUEs was inevitable, which could further lower the prediction accuracy in this study. Large training population and/or high heritability of FHB resistance derived from multiple locations’ phenotypic evaluation could also contribute to the high prediction accuracy observed from previous studies (Mirdita et al., 2015a).

Prediction accuracy of between-crosses or families is generally low because of distant relationship between training population and validation population (Crossa et al., 2014; Herter et al., 2019b). In this study, we found that accuracies of 0.22–0.44 for predicting FHB severity over breeding cycles was not dramatically decreased compared to cross-validation. This could be thanks to the high level of genetic relationships between different breeding cycles, where some elite cultivars and their derived lines were commonly used as parents for crossing blocks. This study aimed to develop an initial prediction model, and will frequently update it by adding newly phenotyped and genotyped breeding lines. In the NDSU HRSW breeding program during the period of this study, over 1,300 inbred breeding lines were evaluated for FHB resistance each year in the FHB field nurseries at two locations in hill plots. This includes around 750 F_7_ lines being tested for the first time, 350 second year F_8_ lines, 150 F_9_ advanced lines, and 75 F_10_ elite lines. Yield tests, quality screening, and selection for other economically important diseases were conducted in each generation. It was reported that genomic assisted selection by combining phenotypic data and predicted values from GS model provided higher selection accuracy than phenotypic selection alone for grain yield and protein content at preliminary yield trial, where phenotypic data is collected from few environments with few or no replications (Michel et al., 2017). We plan to perform genomic assisted selection for FHB resistance at F_7_ generation, where FHB severity will be estimated using both phenotypic data and predicted values from the initial GS model. GS model will be updated by adding the F_7_ lines in the training population. Implementing GS pre-selection of FHB resistance on untested early generation lines like F_6_ lines can be carried out once high prediction accuracy is obtained from the updated prediction model.

One limiting factor to utilize GS in pre-selection of promising lines is high genotyping cost for a large number of selection lines. Similar to other GS studies in wheat breeding populations (Jiang et al., 2015; Haile et al., 2018), we also found that a relatively small number of markers (e.g., 1000 markers) can achieve high prediction accuracy, owing to the high level of linkage disequilibrium. Simulation and empirical studies proved that a panel of low-density markers (50 to 100s of markers) genotyped for inbred progenies and imputed to high-density markers could maintain high prediction accuracies (Hickey et al., 2015; Jacobson et al., 2015; Gorjanc et al., 2017). Such a panel of low-density markers could be genotyped with a SNP array or amplicon GBS at a low cost, potentially enabling the utility of GS on larger populations (Campbell et al., 2015; Pembleton et al., 2016). Together with advances in low-cost genotyping and imputation methods, the high prediction accuracy found in this work pave the way to implement GS for FHB resistance in the NDSU HRSW breeding program.

The 427 breeding lines from the NDSU HRSW breeding program represent the current breeding pool. Few of the new breeding lines showed better FHB resistance than ND2710, one of the first experimental lines released by the NDSU HRSW breeding program from efforts to introduce FHB resistance from Sumai 3 into elite wheat lines adapted to the Northern Great Plains (Frohberg et al., 2004). Multiple target traits including grain yield, end-use quality traits, disease resistance, etc. are selected simultaneously in the NDSU HRSW breeding program, which can partially explain the relatively static FHB resistance development observed in these new breeding lines. The potential to utilize GS pre-selection for multiple traits like FHB resistance, grain yield, and other end-use quality traits at early generations further enhances the advantage conferred by GS compared to traditional phenotypic selection in HRSW variety development.

## Data Availability

All datasets for this study are included in the manuscript and the Supplementary Files.

## Author Contributions

SZ and XL conceived and designed the study. YL, ES, JF, JH, AG, MM, SZ, and XL generated and analyzed the data. All authors wrote and approved the manuscript.

## Conflict of Interest Statement

The authors declare that the research was conducted in the absence of any commercial or financial relationships that could be construed as a potential conflict of interest.

## Supplementary Material

FIGURE S1 Scree plot of the first 30 PCs derived from a principle component analysis.

FIGURE S2 Distribution of the minor allele frequency of the 102,147 SNP markers genotyped for the 439 spring wheat lines.

FIGURE S3 Scatter plot of PC1 and PC2 derived from a principle component analysis for the 439 spring wheat lines.

FIGURE S4 Box plot of identity-by-state similarity among all pairs of breeding lines and pairs of breeding lines between breeding cycles.

TABLE S1 Description of the NDSU hard red spring wheat trials used in this study.

## Abbreviations

BLUP: best linear unbiased predictor
FHB: *Fusarium* head blight
GBS: genotyping-by-sequencing
GS: genomic selection
GWAS: genomic wide association study
HRSW: hard red spring wheat
MAF: minor allele frequency
MAS: marker-assisted selection
NDSU: North Dakota State University
PCA: principle component analysis
rrBLUP: ridge regression best linear unbiased prediction
SNP: single nucleotide polymorphism.
