# Supplementary figures and images for: Genetic Mapping and Prediction Analysis of FHB Resistance in a Hard Red Spring Wheat Breeding Population
Source: Front Plant Sci. 2019 Aug 6;10:1007. doi: 10.3389/fpls.2019.01007 (PMC6691880; doi:10.3389/fpls.2019.01007)

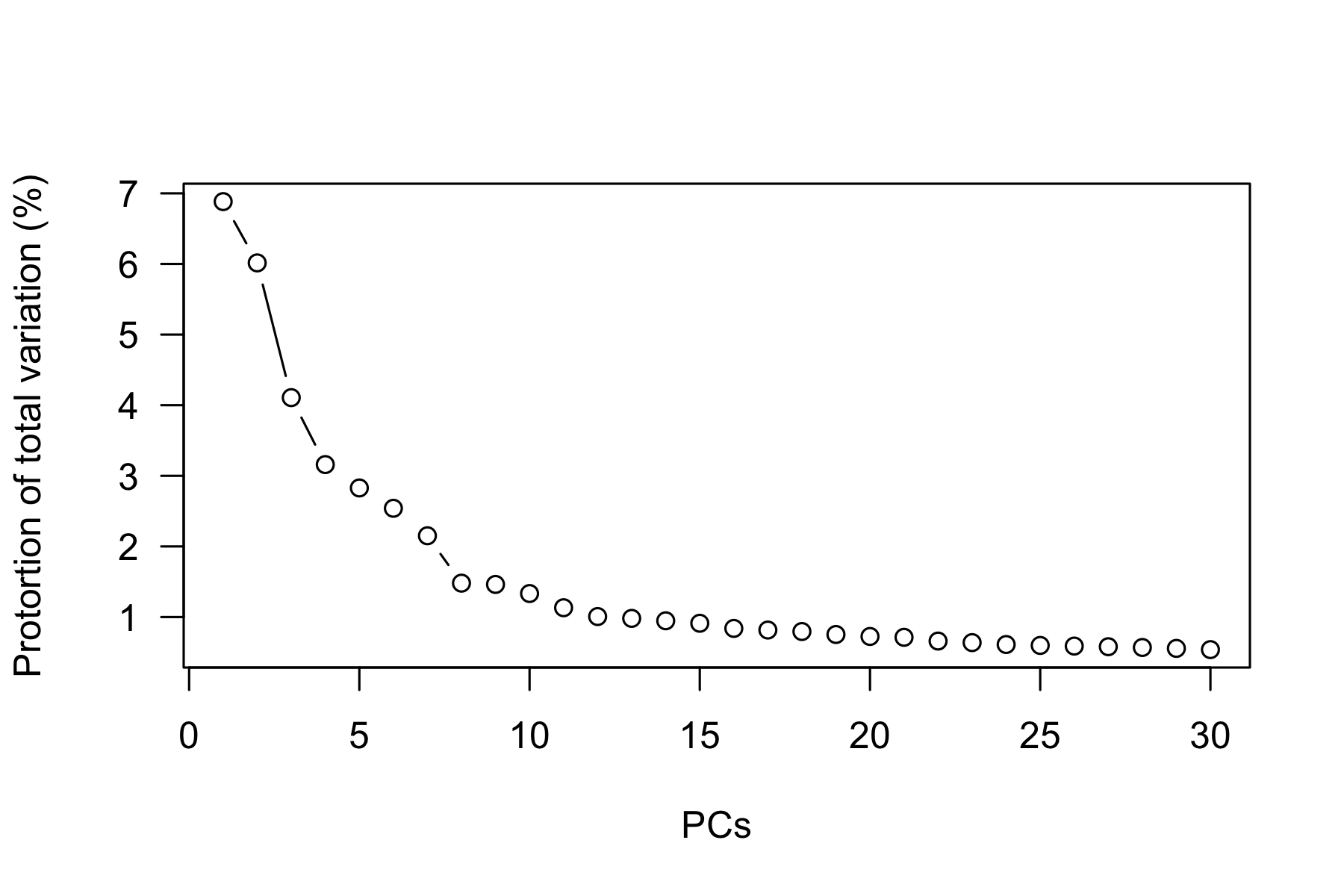

Supplement: FIGURE S1 — Scree plot of the first 30 PCs derived from a principle component analysis. [file Image_1.TIFF]

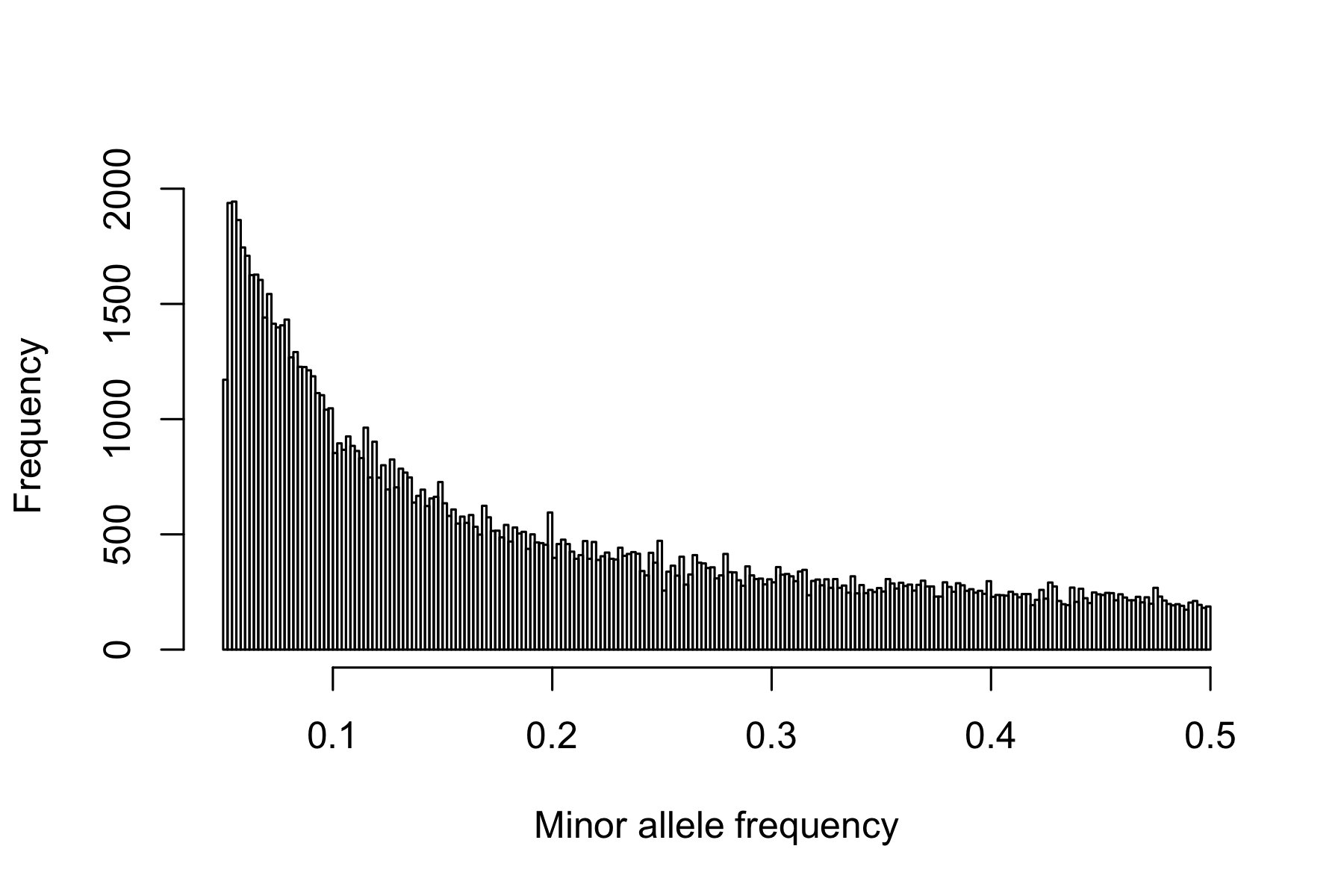

Supplement: FIGURE S2 — Distribution of the minor allele frequency of the 102,147 SNP markers genotyped for the 439 spring wheat lines. [file Image_2.TIFF]

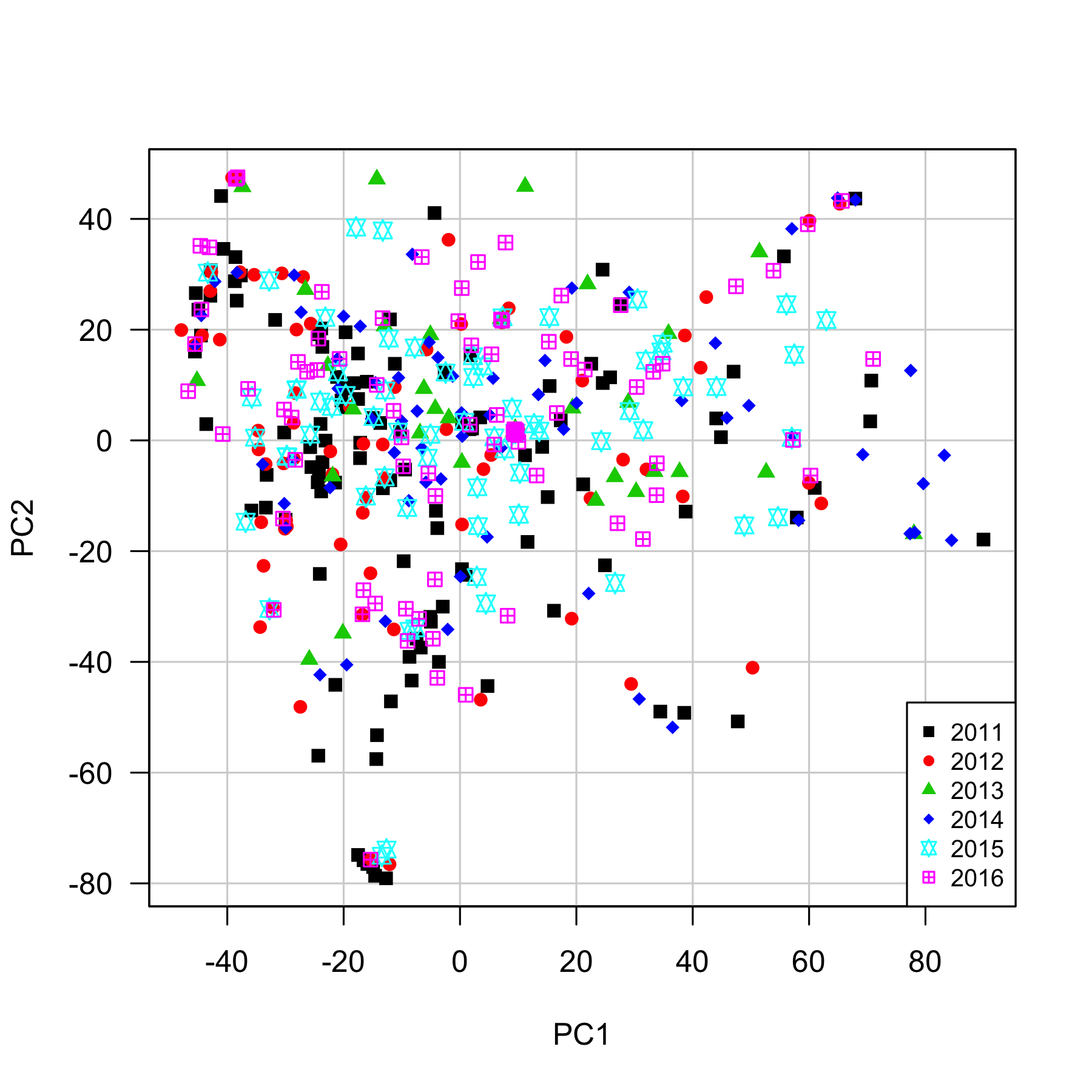

Supplement: FIGURE S3 — Scatter plot of PC1 and PC2 derived from a principle component analysis for the 439 spring wheat lines. [file Image_3.TIFF]

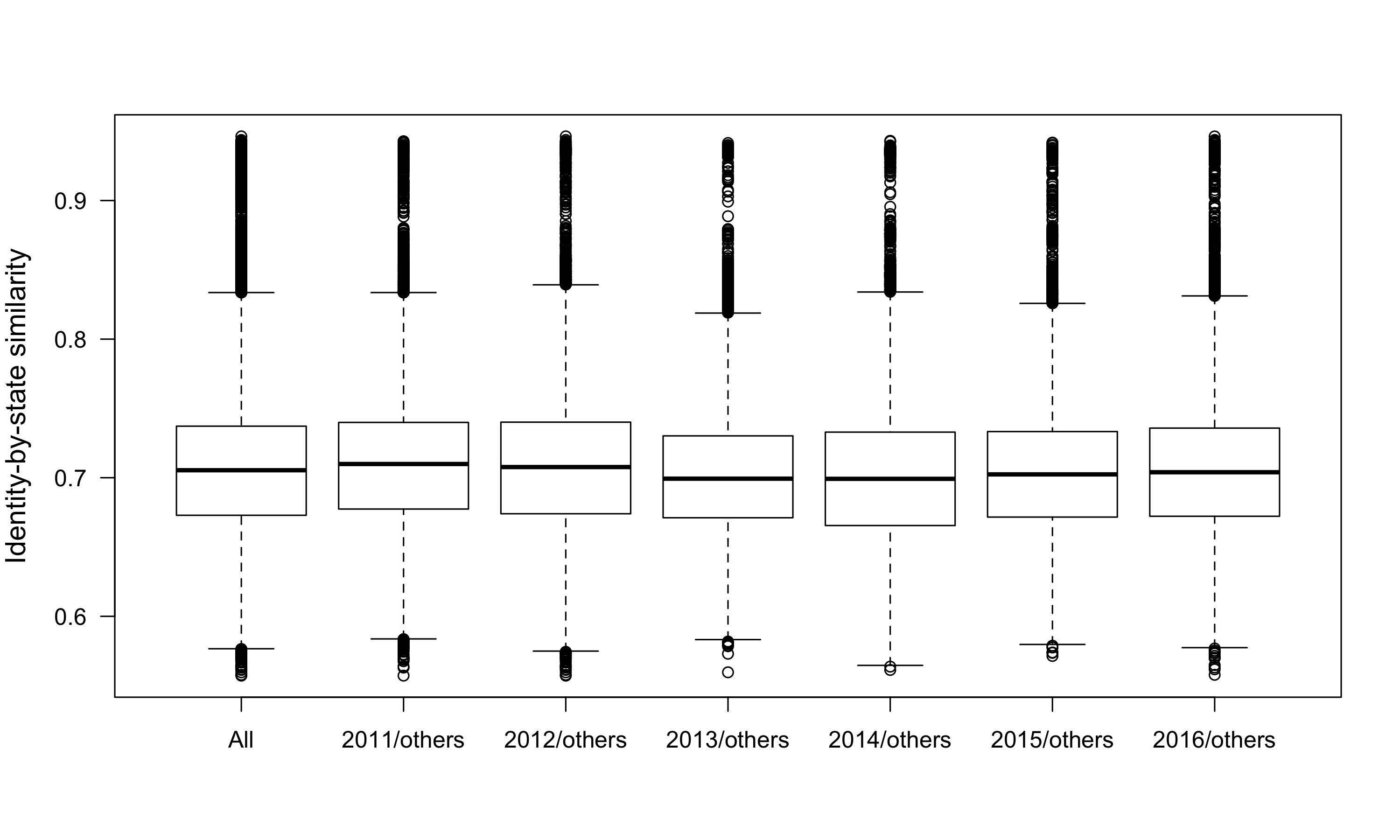

Supplement: FIGURE S4 — Box plot of identity-by-state similarity among all pairs of breeding lines and pairs of breeding lines between breeding cycles. [file Image_4.TIFF]
