# Supplementary material for: Genetic Mapping and Prediction Analysis of FHB Resistance in a Hard Red Spring Wheat Breeding Population
Source: Front Plant Sci. 2019 Aug 6;10:1007. doi: 10.3389/fpls.2019.01007 (PMC6691880; doi:10.3389/fpls.2019.01007)
Supplement: TABLE S1 — Description of the NDSU hard red spring wheat trials used in this study. [file Table_1.DOCX]

Supplementary Table S1. Description of the NDSU hard red spring wheat breeding trials used in this study

| **Location** | **Year** | **Trial** | **Trial name** | **Number of lines** |
| --- | --- | --- | --- | --- |
| Langdon | 2011 | FHB nursery | Langdon11 | 112 |
| Langdon | 2012 | FHB nursery | Langdon12 | 72 |
| Langdon | 2013 | FHB nursery | Langdon13 | 31 |
| Langdon | 2014 | FHB nursery | Langdon14 | 66 |
| Langdon | 2015 | FHB nursery | Langdon15 | 65 |
| Prosper | 2011 | FHB nursery | Prosper11 | 112 |
| Prosper | 2012 | FHB nursery | Prosper12 | 72 |
| Prosper | 2014 | FHB nursery | Prosper14 | 66 |
| Prosper | 2015 | FHB nursery | Prosper15 | 65 |
| Prosper | 2016 | FHB nursery | Prosper16 | 81 |
